# Tickborne Meningoencephalitis, First Case after 19 Years in Northeastern Germany

**DOI:** 10.3201/eid1104.041110

**Published:** 2005-04

**Authors:** Christoph J. Hemmer, Martina Littmann, Micha Löbermann, Michael Lafrenz, Tobias Böttcher, Emil C. Reisinger

**Affiliations:** * University of Rostock Medical School, Rostock, Germany;; † Health Department of the State of Mecklenburg-West Pomerania, Rostock, Germany

**Keywords:** letter, tickborne encephalitis, meningoencephalitis, Northern Germany, Mecklenburg-West Pomerania, FSME

**To the Editor:** Tickborne encephalitis virus (TBEV) is focally distributed in Europe and Asia (between 42° and 63° north latitude). Recently, 5 human tickborne encephalitis cases have been reported, and anti-tickborne encephalitis antibody prevalence in dogs has been observed in southern Norway (*1*). In the last 2 decades, mild winters may have favored a northbound spread and increased tickborne encephalitis incidence (*2*). Tickborne encephalitis is endemic in southern Germany, but no cases had been reported in northeast Germany since 1985 (*3**,**4*).

A 61-year-old man was bitten by a tick at Lake Woblitz, near the town of Neustrelitz in former East Germany, on May 31, 2004, between 8:00 a.m. and 4:00 p.m. The patient's history showed no other tick bites, no stays in tickborne encephalitis–endemic areas, and no tickborne encephalitis vaccination.

On June 9, transient fever and headache developed in the patient, followed 6 days later by difficulty in concentrating, apathy, and a strong urge to sleep. On June 23, the patient was hospitalized with fever (temperature 39.2°C) and mental confusion. Because he had cruised the Nile in December 2003, he was transferred to the Tropical Medicine Division of Rostock University to exclude a diagnosis of malaria. Somnolence, slurred speech, amnestic dysphasia, and impaired fine motor control, but no meningism, focal signs, pyramidal tract, or sensation impairment, were observed. Results of magnetic resonance imaging brain scan and electroencephalogram were normal.

Laboratory tests showed leukocytosis of 9,400 leukocytes/µL and lymphocytopenia of 12%. Alpha-amylase was 254 U/L, lipase 84 U/L, sodium 131 mmol/L, and fibrinogen 4.4 g/L. C-reactive protein and all other routine laboratory parameters were normal.

In the serum, immunoglobulin (Ig)G, but not IgM, was detected against *Borrelia burgdorferi*. Tests of cerebrospinal fluid (CSF) specimens, including polymerase chain reaction (PCR) for herpes simplex virus types 1 and 2, varicella zoster virus, Epstein-Barr virus (EBV), cytomegalovirus, and human herpesvirus 6 were negative. Antibody tests for *Borrelia burgdorferi*, *Mycoplasma pneumoniae*, *Bartonella*, *Brucella*, *Leptospira interrogans*, HIV, EBV, and arboviruses were negative. VDRL (Venereal Disease Research Laboratory) tests, Gram stains, and routine bacterial cultures for common pathogens were also negative (*5*).

Tests on CSF showed mild pleocytosis (9 leukocytes/µL) and high protein concentration (1,322 g/L). Most cells were lymphocytes (89%) and monocytes (10%). Protein analysis showed blood-brain barrier impairment and intrathecal IgM synthesis.

Anti-tickborne encephalitis virus IgG and IgM antibodies were detectable in the serum by enzyme-linked immunosorbent assay 29 days after the tick bite; corresponding CSF titers were borderline. One week later, IgG and IgM antibodies were positive in serum and CSF, while CSF leukocyte count and protein concentration were normal. The anti-tickborne encephalitis serum immunofluorescence titer rose from 1:80 (June 29) to 1:640 (July 14); titers against dengue, West Nile, yellow fever, and Japan B encephalitis were not elevated. Even though CSF specimens were negative for TBEV genome on 2 occasions, a confirmed tickborne encephalitis case had to be reported to the health authorities.

With symptomatic therapy, the patient's condition improved, and he was discharged on July 7 with slight mental slowing. By July 14, he had recovered completely.

Our patient had acquired tickborne encephalitis at a popular tourist site (Figure), where no human cases had occurred for 19 years. From 1959 to 1983, numerous TBEV foci existed in northeastern Germany (*3*). From 1960 to 1985, a total of 4 human cases were seen 10 km east of Neustrelitz. From 1983 to 1989, numerous attempts to cultivate TBEV from ticks or small mammals failed (*3*). In 1992, TBEV genome was detected by PCR in 3 tick pools from the island of Usedom, and in 2 pools from the Darss peninsula, 100 km northeast of Neustrelitz. From 1993 to July 2004, TBEV genome was not detected in 16,098 ticks collected from 275 regions of northeastern Germany, including the county where Lake Woblitz is situated, as part of a statewide surveillance program (State Health Services, unpub. data). However, during 2004, this county reported 24 cases of Lyme disease (2003: 10 cases; 2002: 8 cases; 2001: 1 case). Therefore, our tickborne encephalitis case might represent intensified amplification cycles of tickborne infectious agents in 2004.

**Figure:**
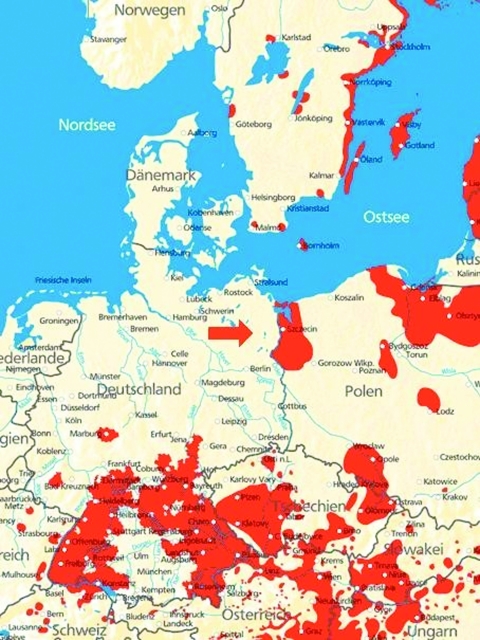
European tickborne encephalitis–endemic areas (in red) and new infection site of case in northeastern Germany (arrow). Map provided by Baxter Germany GmbH.

The absence of tickborne encephalitis cases for 20 years does not likely represent a lack of data before or a lack of interest after the reunification of Germany. Tickborne encephalitis was a reportable disease under East German regulations, and tickborne encephalitis surveillance was intensified after reunification (*3*).

Eight weeks after our patient's tick bite, 160 *Ixodes ricinus* ticks were collected from 10 pools near Lake Woblitz. RNA was isolated in 5 mol/L guanidium isothiocyanate solution, extracted by phenolchloroform, and precipitated with ethanol. cDNA was amplified by nested reverse transcription–PCR and detected by electrophoresis (*6*). In 2 of these pools, PCR directed towards the 5´ terminal noncoding region of the TBEV genome yielded a 104-bp fragment, but the sequence was not specific for flaviviruses.

This case does not prove a northbound spread of tickborne encephalitis in northeastern Germany. Rather, it shows that after years of negative tickborne encephalitis test results in ticks, old tickborne encephalitis foci may retain activity. Thus, tickborne encephalitis should be included in the differential diagnosis of meningoencephalitis in northeastern Germany, even if the patient has not been in tickborne encephalitis–endemic areas.
